# PARP Inhibitors in Cancer Therapy: Magic Bullets but Moving Targets

**DOI:** 10.3389/fonc.2013.00279

**Published:** 2013-11-14

**Authors:** Girish M. Shah, Mihaela Robu, Nupur K. Purohit, Jyotika Rajawat, Lucio Tentori, Grazia Graziani

**Affiliations:** ^1^Laboratory for Skin Cancer Research, CHU-Q (CHUL) Research Centre, Laval University, Quebec City, QC, Canada; ^2^Department of System Medicine, University of Rome “Tor Vergata”, Rome, Italy

**Keywords:** poly(ADP-ribose) polymerase (PARP), PARP inhibitors (PARPi), cancer therapy, BRCA-mutant cancers, synthetic lethality, combination therapy, multiple targets of PARPi

The pharmacological inhibitors of poly(ADP-ribose) polymerase-1 (PARP-1) have reached the first milestone toward their inclusion in the arsenal of anti-cancer drugs by showing consistent benefits in clinical trials against BRCA-mutant cancers that are deficient in the homologous recombination repair (HRR) of DNA double strand breaks (DSB) (1, 2). PARP inhibitors (PARPi) also potentiate therapeutic efficacy of ionizing radiation and some chemotherapeutic agents (1). These effects of PARPi were initially linked to inhibition of the role of PARP-1 in base excision repair (BER) of DNA damaged by endogenous or exogenous agents, resulting in accumulation of single strand breaks (SSB), which upon conversion to toxic DSB lesions would kill cancer cells deficient in DSB repair (1, 3, 4). However, PARPi lethality in HRR-deficient cancers can also be explained by other mechanisms not involving a direct effect of PARPi on BER [reviewed in Ref. (5, 6)]. In addition, therapeutic benefits of PARPi with agents such as carboplatin in HRR-proficient and -deficient tumors [reviewed in Ref. (1, 7)], simply cannot be explained by BER inhibitory effect of PARPi. Therefore, PARPi are like magic bullets that can kill cancer cells under different circumstances, but to comprehend their global scope and limitations, here we discuss the full range of their targets and the possible impact of broad specificity of current PARPi during prolonged therapy of cancer patients.

## Mechanisms of Action of PARPi in Cancer Therapy: Magic Bullets but Moving Targets

It is not surprising that the mechanism of action of PARPi in killing cancer cells still remains an open question, because its principal target PARP-1 is a multifunctional protein implicated in various cellular responses to DNA damage ranging from different pathways of DNA repair and cell death to stress signaling, transcription, and genomic stability (8, 9), all of which could be affected by PARPi and thus influence outcome of cancer therapies. Following are various possibly overlapping mechanisms for the anti-cancer effect of PARPi.

### BER/HRR nexus for synthetic lethality of PARPi in BRCA-mutant cancers

**Figure 1:**
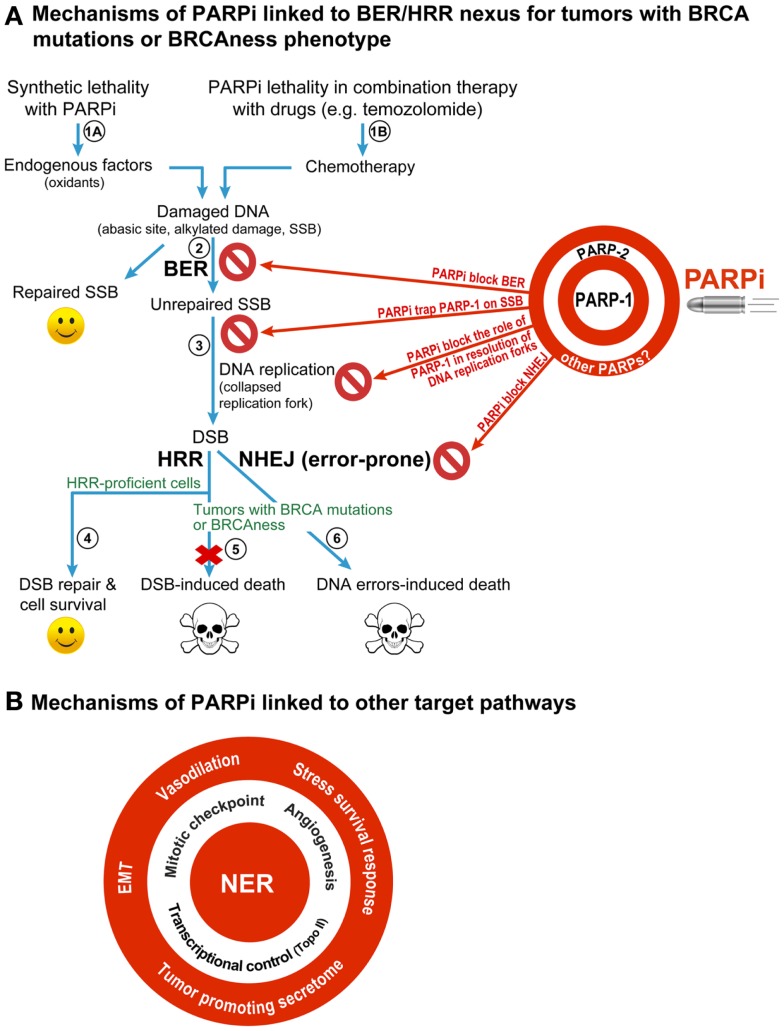
**Different mechanisms for therapeutic efficacy of PARPi in cancers**. **(A)** BER/HRR model: this model focuses on the role of PARP-1, the principal target of PARPi, in BER that removes abasic sites and SSB created constantly in the mammalian genome by endogenous oxidants (steps 1A). During BER, the binding of PARP-1 to SSB leads to stimulation of its catalytic activity of forming polymers of ADP-ribose (PAR) from its substrate NAD^+^. The PAR and PARP-1 interact with and recruit the key BER scaffold protein XRCC1, whereas PAR-modified PARP-1 loses its affinity to bind to SSB and vacates the site for BER to continue. When PARPi suppress the role of PARP-1 in BER (step 2), the unrepaired SSB would accumulate and collapse the DNA replication fork to form potentially lethal DSB (step 3). The normal cells would survive by repairing these DSB by HRR (step 4), but the HRR-deficient BRCA-mutants would die due to unrepaired DSB (step 5) or possibly due to excessive reliance on the error-prone NHEJ repair pathway to remove DSB (step 6). This BER/HRR nexus also explains the effectiveness of combination therapy of PARPi with drugs that cause DNA damage that is repaired by BER (step 1B). Since PARP-2 is also known to play a role in BER, and since current PARPi are also known to inhibit PARP-2, the effect of PARPi may also be mediated by targeting of the functions of PARP-2 in BER, as shown on the target board along with PARP-1. In addition, the inhibitory effect of PARPi on other PARPs could also influence therapeutic efficacy of PARPi (see target board), although their contribution to BER/HRR mediated therapeutic effect of PARPi is not yet fully assessed. **(B)** Other targets of PARPi that can confer therapeutic benefits of PARPi: PARPi could also be effective anticancer agents by targeting the role of PARP-1 in other DNA repair pathways, such as NER; or other cellular pathways, such as control over cell cycle, tumor angiogenesis, transcription, epithelial-mesenchymal transition (EMT), stress survival response, vasodilation, or tumor-promoting secretome.

It was first demonstrated by two teams (3, 4) that two individually non-lethal conditions, i.e., PARPi-mediated inhibition of PARP-1 and BRCA mutation-induced HRR deficiency in cancer cell, would become synthetic lethal when combined in a single cell [reviewed in Ref. (1, 5, 10, 11)] (Figure 1A). This model focuses on the role of PARP-1 in BER, the pathway that repairs abasic sites and SSB that are constantly created in the mammalian genome by endogenous oxidants. When PARPi suppress the role of PARP-1 in BER, the unrepaired SSB would accumulate and collapse the DNA replication fork to form potentially lethal DSB. The normal cells would survive by repairing these DSB by HRR, but the HRR-deficient BRCA-mutants would die due to unrepaired DSB or possibly due to excessive reliance on the error-prone non-homologous end-joining (NHEJ) repair pathway to remove DSB (Figure 1A). This model also covers minor variations of the central theme as reviewed recently (1, 10) (Figure 1A). For example, tumors with other conditions that cause HRR deficiency or “BRCAness” phenotype would also be susceptible to PARPi. It permits inclusion of PARP-2 and its role in BER as target of PARPi, because most current PARPi also inhibit PARP-2 (10). It also explains the potentiating effect of PARPi in the combination therapy with radiation or chemicals, such as temozolomide, irinotecan, or topotecan, because DNA damage caused by these agents is also repaired by BER.

### Alternative targets of PARPi in BRCA-mutant cancers

However, the above mechanism is inadequate to explain all the effects of PARPi seen in BRCA-mutant cancers, which could be explained by the effect of PARPi on alternate targets, as reviewed earlier (5, 6, 10) (Figure 1A). In brief, (i) PARPi could be trapping PARP-1 or PARP-2 to SSB with resultant PARP-SSB complex that would be more toxic than unrepaired SSB or even knockdown of PARPs (5, 12). (ii) PARPi could act via upregulation of NHEJ pathway, which would presumably cause genomic instability and eventual lethality (13). (iii) PARPi could suppress the role of PARP-1 in reactivating DNA replication forks (5). Thus, apart from BER/HRR nexus, there could be NHEJ/HRR or DNA replication/HRR nexus to explain PARPi lethality in BRCA-mutant cancers.

### Expanding universe of potential targets of PARPi

Therapeutic effectiveness of PARPi seen with some drugs cannot be explained by any of the above models, e.g., the potentiating effects of PARPi on the platinum-based drugs such as carboplatin, cisplatin, or oxaliplatin on HRR-deficient or -proficient tumors [reviewed in Ref. (1, 7)] (Figure 1B). These observations were further supported by recent studies showing the potentiating effect of PARPi veliparib on carboplatin treatment of patients with BRCA-mutant breast cancers (14) or carboplatin and phosphoinositide 3-kinase mTOR inhibitor treatment of mouse xenografts of BRCA-competent triple negative breast cancer cells (15). Since platinum compounds cause DNA damage that is largely repaired by the nucleotide excision repair (NER) pathway and not BER, we need to think beyond BER for an explanation. Moreover, BER was shown to mediate toxicity of cisplatin by competing with the repair of cisplatin inter-strand cross-links and DSB caused by these links (16). Therefore, if PARPi effect was mainly via inhibition of BER, we should have observed less and not more toxicity of cisplatin.

One possible explanation is that PARPi could be causing vasodilation (Figure 1B) to improve intra-tumoral delivery of platinum drugs (1), although it needs to be confirmed if this generalized effect could also potentiate other drugs. On the other hand, recently discovered roles of PARP-1 in improving the efficiency of NER-mediated removal of UV-induced DNA damage (17–19) provides a more handy explanation for the PARPi-induced potentiation of platinum compound-based drugs, which also cause DNA damage that is repaired by NER (Figure 1B). This NER targeting effect of PARPi alone can account for death of HRR-proficient tumors, as seen in clinical trials [reviewed in Ref. (1, 7)] and supported by *in vitro* results showing that PARP-1 depletion (20) or inhibition (19) decreases clonogenic survival of UV-exposed human skin fibroblasts with no reported HRR-deficiencies. Of course, PARPi could have an additional effect in this model due to suppression of the role of PARP-1 in HRR pathway (21). In addition, in the PARPi-treated BRCA-mutant HRR-deficient tumors, the unrepaired DNA damage by platinum drugs could collapse the DNA replication fork to form DSB and cause lethality. Thus, the NER effect alone or NER-HRR nexus could be possible explanations for the lethality of PARPi/platinum compounds in HRR-proficient or -deficient tumors.

The clinical and preclinical studies have also revealed other targets of PARPi in cancer therapies that are linked to various roles of their multifunctional target PARP-1 in following cellular processes (Figure 1B). (i) Transcriptional control of drug-target genes: PARPi have been shown to increase toxicity of topoisomerase II-poison doxorubicin *in vitro* (22) or in xenografted tumors in mice (23). This effect could be due to doxorubicin-induced decrease in expression and activity of PARP-1 (24) or PARPi-mediated increase in expression of topoisomerase II, because the transcription activator Sp1 loses its affinity for the topoisomerase II-promoter region upon modification by polymer of ADP-ribose (PAR) created by the activated PARP-1 (22). (ii) Mitotic checkpoint: the beneficial effects of PARPi with microtubule stabilizing mitotic inhibitor paclitaxel in patients with recurrent metastatic gastric cancers with BRCAness phenotype (25) could be linked to suppression of the role of PARP-1 in maintaining the mitotic checkpoint via PARylation of itself or the mitotic checkpoint protein CHFR (26, 27). An abrogation of mitotic checkpoint would kill cancer cells, because they will be forced to divide before resolution of the damage. (iii) Tumor-promoting secretome: PARPi-mediated suppression of the role of PARP-1 in elaborating tumor-promoting secretome containing cytokines and growth factors has been suggested as a cause for decreasing the resistance to another mitotic inhibitor docetaxel (28). (iv) Angiogenesis: the role of PARP-1 in promoting angiogenesis that fuels the growth of tumors can also be target of PARPi, because PARP-1 depletion or PARPi reduce vessel formation (29) and expression of markers of angiogenesis in melanoma (30) or endothelial cells (31). (v) Epithelial-mesenchymal transition (EMT) and metastasis: PARPi or PARP-1 depletion-induced reduction in aggressiveness and growth of metastatic melanoma in animal studies (30, 31) along with decreased markers for EMT (31, 32) suggest that the increase in progression-free survival of PARPi-treated patients could be due to reduction in the proliferation rate of the primary tumor and repression of its metastatic potential. (vi) Stress survival response: finally, cancer cells respond to any therapy by elaborating various stress responses to survive; and PARP-1 and its product PAR play key roles in these stress responses (9). Hence the suppression of pro-survival stress responses could explain the effectiveness of PARPi with any anti-cancer drug. An expanding list of potential targets of PARPi provides us with a much larger vision of the future applications of PARPi in cancer therapy.

## Broad Specificity of PARPi: A Key Issue for the Future of PARPi Therapy

There are two basic issues arising from the broad specificity of current PARPi.

(a) PARPi can inhibit more than one PARP (“they are bazookas not bullets”): many of the current PARPi in clinical trials display strong binding to PARPs 1–4 (33), and inhibit both PARP-1 and 2 at clinically relevant concentrations (10). Most studies assume that the effect of PARPi on both PARP-1 and 2 is important for therapy; however, this may not be the case. In fact, some studies using specific knockdown of PARPs showed that only the knockdown of PARP-1, but not PARP-2, replicates: (i) the synthetic lethal effect of PARPi on BRCA2 mutant cells (3); (ii) potentiation of cisplatin by PARPi in BRCA-proficient triple negative breast cancer cells (34); and (iii) sensitization of melanoma cells *in vitro* to temozolomide (35). On the other hand, the effect of PARPi on gemcitabine in the above breast cancer cells was replicated by PARP-2 knockdown and not PARP-1 knockdown (34). In contrast, the siRNA for PARP-1 could specifically prevent the growth of BRCA-deficient ovarian cancer cell-derived tumors in mice (36). Since the double knockout of PARP-1 and PARP-2 is embryonic lethal (37), we must verify the assumption that gratuitous inhibition of unrelated PARPs has no effect on the end-results.
(b) Indiscriminate inhibition of all the roles of a given PARP by PARPi (“we are nuking the entire PARP-landscape”): PARP-1, the principal target of PARPi, is a multifunctional protein that is implicated not only in DNA repair but also in various forms of cell death, transcription, epigenetic control of gene expression, and chromatin remodeling (8, 38). Hence even if we were to develop novel PARPi to specifically inhibit only PARP-1, it will still shut down most if not all the functions of PARP-1. Similar arguments can be made for PARPi-mediated suppression of different roles of PARP-2. Although adverse genomic consequences of PARPi therapy have not yet been reported, we need to consider that prolonged PARPi therapy may cause genome instability because PARP-1^−/−^ mouse embryonic fibroblasts have a tendency to become tetraploid (39, 40), and the susceptibility of PARP-1^−/−^ female mice to develop mammary carcinoma is enhanced if p53 is also mutated, a phenomenon frequently observed in cancers (41). In effect, PARPi are the magic bullets, but instead of doing precision targeting with them for the desired effect, we are simply nuking the entire spectrum of functions of that target PARP, which could result in unintended consequence during maintenance (prolonged) therapy with PARPi including survival of damaged cancer cells, development of secondary tumors as a consequence of genomic instability and resistance to PARPi. Thus, while the current broad specificity PARPi work properly for short-term cancer therapy, there is a need for development of new and more specific PARPi that are unique not only for a given PARP but also for a given function of that PARP related to its anti-cancer effect.

It is heartening that PARPi have shown some clinical benefit for BRCA-mutant cancer patients in clinical trials as monotherapy or as a combination therapy, but we need to do a lot more to understand the therapeutic effect of PARPi to establish them firmly in the arsenal of anti-tumor agents against variety of cancers.
